# Human Infections with Borna Disease Virus 1 (BoDV-1) Primarily Lead to Severe Encephalitis: Further Evidence from the Seroepidemiological BoSOT Study in an Endemic Region in Southern Germany

**DOI:** 10.3390/v15010188

**Published:** 2023-01-09

**Authors:** Markus Bauswein, Lisa Eidenschink, Gertrud Knoll, Bernhard Neumann, Klemens Angstwurm, Saida Zoubaa, Markus J Riemenschneider, Benedikt M J Lampl, Matthias Pregler, Hans Helmut Niller, Jonathan Jantsch, André Gessner, Yvonne Eberhardt, Gunnar Huppertz, Torsten Schramm, Stefanie Kühn, Michael Koller, Thomas Drasch, Yvonne Ehrl, Bernhard Banas, Robert Offner, Barbara Schmidt, Miriam C. Banas

**Affiliations:** 1 Institute of Clinical Microbiology and Hygiene, University Hospital Regensburg, 93053 Regensburg, Germany; 2 Department of Neurology, Donau-Isar-Klinikum Deggendorf, 94469 Deggendorf, Germany; 3 Department of Neurology, University of Regensburg, Bezirksklinikum, 93053 Regensburg, Germany; 4 Department of Neuropathology, University Hospital Regensburg, 93053 Regensburg, Germany; 5 Regensburg Department of Public Health, 93059 Regensburg, Germany; 6 Department of Epidemiology and Preventive Medicine, University of Regensburg, 93053 Regensburg, Germany; 7 Institute of Medical Microbiology and Hygiene, University of Regensburg, 93053 Regensburg, Germany; 8 Institute for Medical Microbiology, Immunology and Hygiene, University Hospital Cologne and Faculty of Medicine, University of Cologne, 50935 Cologne, Germany; 9 Center for Clinical Studies, University Hospital Regensburg, 93053 Regensburg, Germany; 10 Department of Nephrology, University Hospital Regensburg, 93053 Regensburg, Germany; 11 Institute of Clinical Chemistry and Laboratory Medicine, Department of Transfusion Medicine, University Hospital Regensburg, 93053 Regensburg, Germany

**Keywords:** Borna disease virus 1 (BoDV-1), epidemiology, encephalitis, diagnostics, ELISA, indirect immunofluorescence assay (iIFA), solid organ transplantation, linear epitope mapping, molecular mimicry, endogenous Borna-like elements

## Abstract

More than 40 human cases of severe encephalitis caused by Borna disease virus 1 (BoDV-1) have been reported to German health authorities. In an endemic region in southern Germany, we conducted the seroepidemiological BoSOT study (“BoDV-1 after solid-organ transplantation”) to assess whether there are undetected oligo- or asymptomatic courses of infection. A total of 216 healthy blood donors and 280 outpatients after solid organ transplantation were screened by a recombinant BoDV-1 ELISA followed by an indirect immunofluorescence assay (iIFA) as confirmatory test. For comparison, 288 serum and 258 cerebrospinal fluid (CSF) samples with a request for tick-borne encephalitis (TBE) diagnostics were analyzed for BoDV-1 infections. ELISA screening reactivity rates ranged from 3.5% to 18.6% depending on the cohort and the used ELISA antigen, but only one sample of a patient from the cohort with requested TBE diagnostics was confirmed to be positive for anti-BoDV-1-IgG by iIFA. In addition, the corresponding CSF sample of this patient with a three-week history of severe neurological disease tested positive for BoDV-1 RNA. Due to the iIFA results, all other results were interpreted as false-reactive in the ELISA screening. By linear serological epitope mapping, cross-reactions with human and bacterial proteins were identified as possible underlying mechanism for the false-reactive ELISA screening results. In conclusion, no oligo- or asymptomatic infections were detected in the studied cohorts. Serological tests based on a single recombinant BoDV-1 antigen should be interpreted with caution, and an iIFA should always be performed in addition.

## 1. Introduction

Infections with Borna disease virus 1 (BoDV-1) cause severe cases of encephalitis in humans. After the identification of variegated squirrel bornavirus 1 (VSBV-1, species *Mammalian 2 orthobornavirus*) in an encephalitis cluster associated with the breeding of variegated squirrels in 2015 [1], BoDV-1 (species *Mammalian 1 orthobornavirus*) was the second member of the *Bornaviridae* family (genus *Orthobornavirus*) whose zoonotic potential was proven in 2018 [2,3] after decades of controversy about possible associations with human cases of psychiatric disorders [4]. The bicolored white-toothed shrew (*Crocidura leucodon*) was identified as a reservoir of BoDV-1 [5,6]. Long before the unequivocal confirmation of human infections, Borna disease has well been known to affect horses, sheep, and goats in the endemic areas of southern and eastern Germany, Austria, Liechtenstein, and Switzerland. As causative agent of Borna disease, BoDV-1 was identified in 1990 as an enveloped non-segmented RNA virus with an 8.9 kbp genome in negative polarity that encodes six structural proteins [7,8]. The glycosylated membrane protein (G) mediates viral entry by binding to a presently unknown receptor [9]. The M protein is a glycosylated matrix protein [10], while the nucleocapsid protein (N) binds to viral RNA and forms the ribonuclear particle (RNP) together with the phosphoprotein (P) and large protein (L; RNA-dependent RNA-polymerase) [11]. The accessory X protein has regulative functions [12]. BoDV-1 replicates within the cell nucleus and leads to persistent infections [8]. In spill-over hosts, severe clinical symptoms are attributed to an immunopathology, driven by a dysregulated inflammation and a presumably T-cell-mediated tissue destruction [13,14,15]. After initial unspecific symptoms, the clinical course of the disease is characterized by a rapid onset of severe neurological symptoms including memory loss, seizures, apnea, and deep coma. Case fatality rates are very high (>90%). No proven curative treatment has been established so far, although off-label treatment approaches, e.g., with the antiviral drugs ribavirin and favipiravir, have been attempted [16]. Since the description of the first cases, more than 40 human infections associated with severe encephalitis have been reported to German health authorities. Most patients lived in the southern German state of Bavaria, but cases from the north and east of Germany have also been described [17,18,19,20,21,22]. As symptoms may resemble other severe neurological disorders such as Miller Fisher syndrome, Guillain-Barré syndrome, encephalopathy, or autoimmune encephalitis, diagnosis is not easy and the underreporting of cases is likely. Especially in the initial stages or even during the whole disease period, cell counts in cerebrospinal fluid (CSF) can be normal or only slightly elevated [17,21], leading to difficulties with the timely initiation of diagnostic steps.

There is only limited data whether human BoDV-1 infections, apart from the severe cases of encephalitis outlined above, can lead to oligo- or even asymptomatic courses of disease. In one of the first described case series, BoDV-1 was transmitted from an organ donor without neurological symptoms, who had died of sudden cardiac arrest, to three organ recipients [2].

With the seroepidemiological BoSOT study (“BoDV-1 after solid-organ transplantation”), we addressed the question of whether there are undetected oligo- or asymptomatic infections in humans. Because at least five of the confirmed human infections to date have involved solid organ recipients [2,18], a particular focus of the study was on patients with this characteristic.

## 2. Materials and Methods

### 2.1. Study Cohorts

The BoSOT study was conducted in an endemic region of southern Germany with recently confirmed BoDV-1 infections in humans and animals [18,21]. The study was approved by the Ethics Committee of the Faculty for Medicine, University of Regensburg, Regensburg, Germany (reference number: 21-2202-101) and registered in the DRKS trial register (DRKS00025180) on 4 May 2021. As participants, 216 healthy blood donors at the Institute of Clinical Chemistry and Laboratory Medicine, Department of Transfusion Medicine, University Hospital Regensburg, Regensburg, Germany were included in the study from 2 November 2021 to 18 May 2022. The Department of Nephrology at the University Hospital Regensburg enrolled 280 outpatients at routine follow-up after solid organ transplantation from 21 May 2021 to 12 January 2022. All study participants of the BoSOT study (all aged ≥18 years) were included after informed consent. Blood samples for research purposes were drawn as part of a routine venipuncture, either for the purpose of a blood donation (blood donors) or for the routine check-up (transplant patients). No additional venipunctures for research purposes only were performed. The serum of all participants was screened for anti-BoDV-1-IgG antibodies. Furthermore, participants were asked to answer a questionnaire (Table S1). Case report forms were electronically collected and stored on the web-based clinical data management system REDCap in cooperation with the Center for Clinical studies at the University Hospital Regensburg.

In addition to the BoSOT study cohorts, all serum/plasma or CSF samples sent to the section of virology at the Institute of Clinical Microbiology and Hygiene, University Hospital Regensburg, Regensburg, Germany, between 10 May 2021 and 28 July 2022 with a request for tick-borne encephalitis (TBE) diagnostics were serologically tested (serum/plasma samples) or tested by RT-qPCR (CSF samples) for BoDV-1 as part of the diagnostic procedure.

As a control cohort for the serological epitope mapping, samples of patients with brain biopsies that tested negative for BoDV-1 by RT-qPCR and serum/plasma samples that tested negative for anti-BoDV-1-IgG by an indirect immunofluorescence assay (iIFA) and ELISA were used [18,21].

The retrospective examination of clinical samples of patients with encephalitis for the detection of new viruses such as BoDV-1 was approved by the Ethics Committee of the Faculty for Medicine, University of Regensburg, Regensburg, Germany (reference number: 18-1248-101).

### 2.2. BoDV-1 ELISA

A BoDV-1 ELISA system using three different recombinant BoDV-1 proteins was performed as previously described [21]. Sequences for the BoDV-1 nucleoprotein (N), X protein, and phosphoprotein (P) were codon-optimized for expression in *E. coli* and cloned into the expression vectors pET30a, pGEX6P, and pET82, respectively. His-tagged N and P protein as well as GST-tagged X protein were purified by chromatography. All proteins were synthesized and purified in cooperation with Mikrogen, Neuried, Germany. Recombinant BoDV-1 proteins diluted in DPBS (Gibco, Thermo Fisher Scientific, Waltham, MA, USA) were used to coat Nunc MaxiSorp plates (Thermo Fisher Scientific, Waltham, MA, USA). N and X proteins were coated at 800 ng/well (in 100 μL of DPBS), P protein at 150 ng/well (in 100 μL of DPBS) at 4 °C overnight and then blocked with 200 μL of 5% fat-free milk powder (Heirler, Radolfzell, Germany) in DPBS with 0.1% Tween 20 (Caelo, Hilden, Germany) at room temperature (RT) for 1 h. After washing three times with 200 μL of DPBS containing 0.1% Tween 20 (DPBS-T), 100 μL of diluted serum/plasma samples were added. All samples were diluted at 1:100 in 1% fat-free milk powder in 0.1% DPBS-T. Plates were incubated at RT for 1 h and then washed nine times with 200 μL of 0.1% DPBS-T. Subsequently, 50 μL of horseradish peroxidase (HRP)-conjugated polyclonal rabbit anti-human IgG (Agilent, Santa Clara, CA, USA) diluted at 1:5000 in 0.1% DPBS-T were added as secondary antibody. After 1 h of incubation at RT, plates were washed nine times with 200 μL of 0.1% DPBS-T. Afterwards, 50 μL of the substrate solution (TMB) (Mikrogen, Neuried, Germany) were added to each well. Samples were incubated at RT for 4 min before adding 50 μL of stop solution (24.9% phosphoric acid) (Mikrogen, Neuried, Germany). After 5 min, the optical density (OD) was determined at 450 nm (OD450) and 630 nm (OD630) in three technical replicates using a plate reader (Microplate Reader Model 680, Bio-Rad, Hercules, CA, USA). For evaluation, the OD630 background values were subtracted from the OD450 values. The OD of the blank was subtracted from all OD values. To control for inter-assay variability, all sample values were normalized to an external standard sample. Cut-offs for each ELISA system were determined using serum samples from 24 patients that had been tested negative by an iIFA (IgG) and/or whose brain tissue had been tested negative by RT-qPCR analysis [18]. Cut-offs were calculated by the arithmetic mean plus 6 standard deviations. The previously assessed sensitivity of the ELISA system in terms of reactivity against at least one BoDV-1 antigen (N, X or P) was 100% for samples with an iIFA titer ≥100 [21].

### 2.3. BoDV-1 iIFA

An iIFA was performed as previously described [2]. Briefly, Vero cells persistently infected with BoDV-1 were mixed at a 1:2 ratio with uninfected Vero cells and cultured overnight in 96-well microtiter plates (Ibidi, Gräfelfing, Germany) to achieve confluent cell layers. Wells with uninfected Vero cells served as negative controls. After the removal of the supernatant, plates were dried for 2 h and then fixed at 80 °C for 2 h. Thereafter, heat-inactivated serum/plasma samples were added in a 2-fold dilution series in TRIS buffer (Sigma-Aldrich, St. Louis, MO, USA) (1:20, 1:40, 1:80). After incubation for 1 h, plates were washed three times with DPBS and incubated with a 1:200 dilution of Cy-3-conjugated polyclonal rabbit anti-human IgG antibody (Jackson ImmunoResearch, West Grove, PA, USA) for 1 h. After a final washing step, the assays were analyzed using fluorescence microscopy. Samples with characteristic fluorescing spots in the nuclei of BoDV-1-infected Vero cells were considered positive. When similar fluorescence signals were detected in non-infected and BoDV-1-infected Vero cells, samples were considered unspecific. All samples were separately evaluated by two trained and experienced members of laboratory staff.

### 2.4. BoDV-1 Immunoblot

For immunoblots, an SDS-PAGE analysis was performed using a 5% polyacrylamide stacking and 12% polyacrylamide separating gel. Lanes were loaded with 100 ng of N protein, 200 ng of X protein, and 100 ng of P protein. After separation, proteins were blotted onto a 0.2 μg nitrocellulose membrane (Amersham Protran Premium 0.2 μm, Thermo Fisher Scientific, Waltham, MA, USA) using a wet tank transfer system. Membranes were blocked with Roti Block (containing Tween 20) (Roth, Karlsruhe, Germany) and then stained with a 1:100 dilution of the serum/plasma samples. A HRP-conjugated polyclonal rabbit anti-human IgG antibody (Agilent, Santa Clara, CA, USA) diluted 1:3000 was used as a secondary antibody. Proteins were detected by chemiluminescence.

### 2.5. BoDV-1 RT-qPCR

RNA extraction and a BoDV-1 specific RT-qPCR were performed following a recently published protocol [2,21]. BoDV-1 RNA was detected using two real-time RT-qPCR assays (mix 1 for BoDV-1 *X/P* gene and mix 6 for *M/G* gene). The in vitro-transcribed RNA molecules were used as standards for quantification.

### 2.6. Serological Linear Epitope Mapping

For a serological linear epitope mapping, a macroarray was used. Cellulose membranes containing spots of 15-mer peptides spanning the whole BoDV-1 N, X, or P protein with an overlap of 11 amino acids were purchased from peptides&elephants (Hennigsdorf, Germany). As a first step, all membranes were moistened with 35% ethanol and then washed three times with 0.05% DPBS-T. Membranes containing N protein-derived peptides were blocked with 5% fat-free milk powder (diluted in 0.05% DPBS-T), while X and P membranes were blocked with BSA (Biomol, Hamburg, Germany) (1 g in 20 mL of 0.05% DPBS-T) at RT for 2 h. Before the first use of each membrane and before the application of new samples, unspecific staining of the secondary antibody was checked. Samples were diluted at a 1:100 ratio in Roti block. Membranes were incubated in diluted samples at 4 °C overnight. After three washing steps in 0.05% DPBS-T for 10 min each, the membranes were incubated with the secondary antibody at RT for 2 h. A HRP-conjugated monoclonal mouse anti-human IgG-Fc antibody (Abcam, Cambridge, UK) was used in a 1:5000 dilution in Roti block as a secondary antibody. After three washing steps in 0.05% DPBS-T for 10 min each, spots were developed for 5 min and subsequently detected by chemiluminescence. To strip the membranes, they were incubated in 8 M of urea (AppliChem, Darmstadt, Germany) containing 1% SDS (Merck, Darmstadt, Germany) at 37 °C overnight. After two washing steps with H_2_O, the membranes were incubated in 60% acetic acid, 30% ethanol, and 10% H_2_O at RT for 1 h. Three washing steps with DPBS were performed before the membranes were washed twice with H_2_O and three times with 30% methanol. Afterwards, the membranes were dried at 37 °C for 45 min and, if not in further use, were then stored at −20 °C. For the spot analysis, the protein array analyzer plug-in (from Carpentier, G. (2010); available online at http://rsb.info.nih.gov/ij/macros/toolsets/Protein Array Analyzer.txt) for ImageJ software was used (URL accessed on 10 May 2022). Automated linear background subtraction (setting: radius for 2D rolling ball = 25) was performed. Furthermore, the background was subtracted as the maximum intensity of additionally measured, non-peptide-bearing regions on the membranes. All intensities were log 2 transformed. Non-defined values (below background) were set to 0. An arbitrary cut-off of 13.5 was established in order to minimize unspecific staining for samples of the negative control. For further analysis, values below the cut-off were set to 0.

### 2.7. Statistical Analysis

Case report forms of the study participants were electronically stored on the web-based clinical data management system REDCap in cooperation with the Center for Clinical Studies at the University Hospital Regensburg, Regensburg, Germany. Data reports were exported as CSV files. The data were analyzed using R software, version 4.0.3 (The R Foundation for Statistical Computing, Vienna, Austria) and GraphPad Prism, version 9.4.1 (GraphPad Software, San Diego, CA, USA). Figures were created using GraphPad Prism.

## 3. Results

### 3.1. Description of Cohorts

Three different cohorts were used to screen for BoDV-1-specific antibodies. First, 216 healthy blood donors were recruited by the Institute of Clinical Chemistry and Laboratory Medicine, Department of Transfusion Medicine, University Hospital Regensburg, between November 2021 and May 2022. A total of 34% of participants were female, and the median age was 28 years (IQR: 25–37). Second, 280 outpatients with a median follow-up of 8 years (IQR: 4.0–12.1) after solid organ transplantation were included by the Department of Nephrology, University Hospital Regensburg, between May 2021 and January 2022. Most of the patients (91%) had received a kidney transplantation, 36% were female, and the median age was 60 years (IQR: 52–67). As a third cohort, all samples sent to the Institute of Clinical Microbiology and Hygiene, University Hospital Regensburg between May 2021 and July 2022 with the request of tick-borne encephalitis (TBE) diagnostics were also tested for a BoDV-1 infection for diagnostic purposes. A total of 288 serum or plasma samples of independent patients were serologically tested, while a corresponding CSF sample was available in 258 cases and was examined by RT-qPCR analysis. Consecutive samples of patients were removed from the analysis. The median age of patients was 57 years (IQR: 40–66), and 47% of patients were female. Further characteristics of cohorts are provided in Table 1.

### 3.2. Serological Screening by a BoDV-1 ELISA

**Figure 1:**
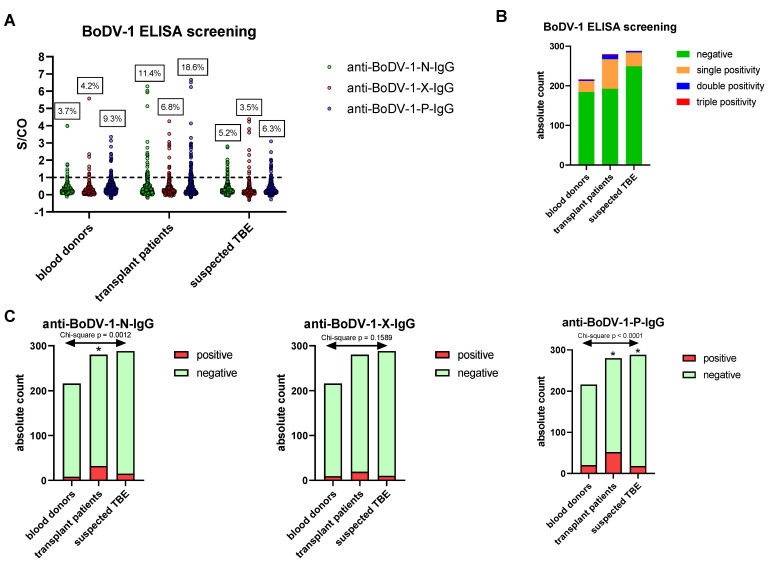
BoDV-1 screening ELISA. Three cohorts of healthy blood donors (n = 216), outpatients after solid organ transplantation (n = 280), and patients with requested TBE diagnostics (n = 288) were screened for anti-BoDV-1-IgG antibodies by an ELISA system using recombinant viral nucleocapsid (N), X, or phosphoprotein (P) as antigens. (**A**) shows the signal-to-cut-off ratios (S/CO) for individual samples and percentages of reactive samples for each cohort and antigen. Fractions of samples with IgG antibodies directed against none (negative), one (single positivity), two (double positivity), or three antigens (triple positivity) are depicted in (**B**). (**C**) shows the absolute counts of negative and reactive samples for each group and antigen. The variables “cohorts” and “test outcome” were statistically dependent for anti-BoDV-1-N-IgG (*p* = 0.0012) and anti-BoDV-1-P-IgG (*p* < 0.0001) based on chi-square tests. As post hoc test, the *p*-values of residuals were analyzed using Bonferroni correction. Residuals were significantly elevated for outpatients after solid organ transplantation (anti-BoDV-1-N-IgG, anti-BoDV-1-P-IgG) and significantly diminished for patients with the requested TBE diagnostics (anti-BoDV-1-P-IgG), as indicated by an asterisk.

As a serological screening test, an in-house BoDV-1 ELISA system using the recombinant viral nucleocapsid (N), X, or phosphoprotein (P) was performed as previously described [21]. Reactivity against a single viral antigen was assumed for values above the previously established cut-off for each antigen (signal-to-cut-off ratio (S/CO) > 1). In the cohort of healthy blood donors, 3.7% (95% CI: 1.9–7.1%) of samples were reactive with regard to the anti-N-IgG ELISA, while reactivity rates were 4.2% (95% CI: 2.2–7.7%) for the anti-X-IgG ELISA and 9.3% (95% CI: 6.1–13.9%) for the anti-P-IgG ELISA, respectively (Figure 1A). In most of the cases, reactivity was directed against a single antigen (13.0% of all samples with 95% CI 9.1–18.1%), while 1.4% (95% CI: 0.4–4.0%) of all samples reacted against two antigens and only 0.5% (95% CI: 0.0–2.6%) against three antigens (Figure 1B). For the cohort of patients with suspected TBE infection, the reactivity rates were similar, reaching values of 5.2% (95% CI: 3.2–8.4%) for anti-N-IgG, 3.5% (95% CI: 1.9–6.3%) for anti-X-IgG and 6.3% (95% CI: 4.0–9.7%) for anti-P-IgG, whereby 12.2% (95% CI: 8.9–16.4%) of all samples were reactive against a single antigen, 1.4% (95% CI: 0.5–3.5%) against two antigens, and 0.0% (95% CI: 0.0–1.3%) against three antigens. Within the cohort of outpatients after solid organ transplantation, however, reactivity rates tended to be higher for all viral antigens. Chi-square tests confirmed significant differences between the cohorts for anti-N-IgG (*p*-value = 0.0012) and for anti-P-IgG (*p*-value < 0.0001) with reactivity rates in the transplant cohort of 11.4% (95% CI: 8.2–15.7%) and 18.6% (95% CI: 14.5–23.5%), respectively, whereas anti-X-IgG positivity rates (transplant cohort: 6.8%, 95% CI: 4.4–10.4%) were not significantly different (Figure 1C).

### 3.3. IIFA Identifies One BoDV-1-Positive Sample

All samples with at least one positive reaction against a viral antigen in ELISA were re-examined by an iIFA.

With regard to the cohorts of healthy blood donors and outpatients after solid organ transplantation, none of the re-tested samples showed a specific nuclear staining pattern in iIFA testing. As a consequence, the reactive ELISA screening results were interpreted as false-reactive.

Within the cohort of patients with suspected TBE-virus infection, however, one sample with single anti-P-IgG reactivity (S/CO = 1.55) was confirmed to be positive for anti-BoDV-1-IgG by iIFA (titer 1:40). The sample belonged to a 50–60-year-old patient (exact age and sex not revealed due to ethical requirements) with a three-week-history of a sudden-onset of severe neurological symptoms. Past medical history was unremarkable. The patient lived in a rural village within the endemic area and worked as a landscaper. Due to cranial nerve involvement which was accompanied by an initially normal cell count in CSF but elevated CSF protein, Miller Fisher syndrome was assumed before initiating encephalitis diagnostics when CSF cell count was mildly increasing (CSF leucocyte cell count 17/µL). The corresponding CSF sample was tested positive by an RT-qPCR test (<300 copies/mL). Post mortem tissue samples with high RNA copies of BoDV-1 confirmed the diagnosis of fatal BoDV-1 infection (RNA copies >10^8^/mL tissue homogenate in sodium chloride for samples from the frontal cortex, temporal cortex, olfactory bulb, mesencephalon, and pons).

All other samples with a reactive screening ELISA within this cohort tested negative by iIFA, and all available CSF samples were negative in the RT-qPCR tests.

### 3.4. Reproducibility of ELISA and Immunoblots

In order to elucidate possible mechanisms for the false reactivity rate of our ELISA screening test, as a first step the ELISA was repeated twice with initially positive samples of the cohorts of healthy blood donors and transplant patients. For anti-N-IgG, 83% of initially positive samples re-tested positive at least once, and 68% were positive in both repetitions. The reproducibility rate (defined as reactivity in both repetitions) for reactive anti-X-IgG samples was 75% (82% reactivity in at least one repetition) and 47% for samples with reactive anti-P-IgG (78% reactivity in at least one repetition). Logistic regressions showed that the probability of reproducibility had the tendency to increase with higher initial S/CO values (slope statistically different from 0 for anti-P-IgG, not statistically significant for anti-N-IgG or anti-X-IgG) (Figure S1).

**Figure 2:**
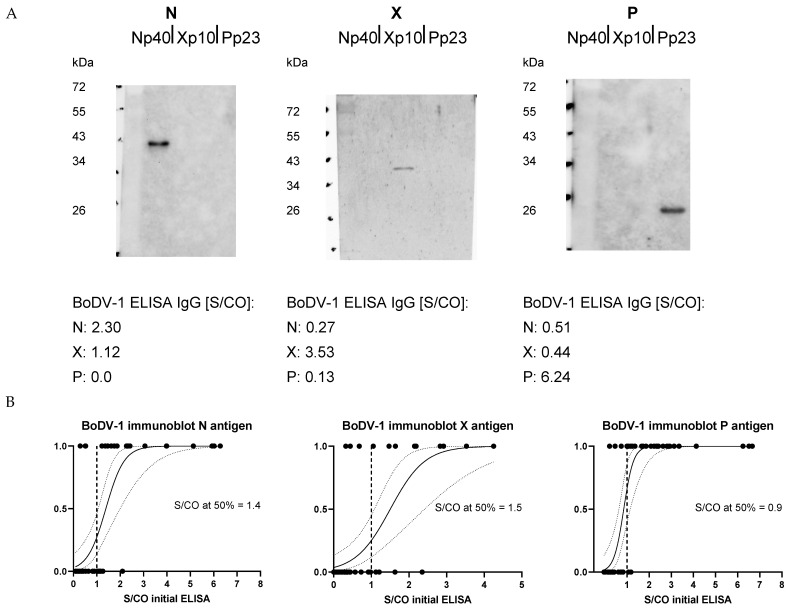
BoDV-1 immunoblots of selected samples with reactive ELISA results. Immunoblots were performed using the same recombinant viral proteins as used for ELISA. Under (**A**), confirmations of antigen-directed IgG antibodies by immunoblots are shown for Np40 protein (His-tagged) on the left, for Xp10 protein (tagged with a 26kDa GST-tag) in the middle, and for Pp23 (His-tagged) on the right. The values given below represent ELISA screening results of the corresponding sample. The probability of an immunoblot band increased with higher S/CO values of the screening ELISA for all used antigens (**B**). Shown are the best-fit lines for logistic regression (0 = no immunoblot band, 1 = immunoblot band) with 95% confidence intervals. Vertical dotted lines represent the ELISA cut-off.

For a selection of samples with highest S/CO values that were identified as reactive by the initial ELISA, immunoblots using the identical recombinant viral proteins were performed (Figure 2A). For 68% of the selected samples, there was a positive N band in the immunoblot when the initial ELISA was positive for anti-N-IgG. Immunoblot re-detection rates for anti-X-IgG and anti-P-IgG were 71% and 93%, respectively. As for reproducibility in ELISA, the probability of a positive band in immunoblot increased with higher initial ELISA S/CO values (Figure 2B).

The sample of the patient correctly diagnosed as BoDV-1 positive was fully reproducible in two ELISA repetitions with respect to a positive anti-P-IgG value and showed a positive P band in the immunoblot assay, while anti-N-IgG and anti-X-IgG were both negative in the ELISA and immunoblot analyses.

Although these results showed limitations in the reproducibility of the ELISA data especially for anti-P-IgG, in general we assumed that the false-reactive results were based on the recombinant viral proteins themselves as immunoblots showed a high concordance with screening ELISA data. Specific bands of the expected molecular weights rendered contaminations with *E. coli* proteins from the expression system an unlikely explanation.

### 3.5. False-Reactive ELISA Results Not Associated with Epidemiological or Clinical Characteristics

In a further step, we addressed the question of whether there was an association with false-reactive ELISA results with epidemiological or clinical characteristics as they were primarily provided for the cohorts of healthy blood donors and transplant patients by a questionnaire (Table S1).

For all three cohorts, false reactivity rates of the ELISA were not significantly associated with sex (Table 2). Within the groups of healthy blood donors and transplant patients, false-reactive ELISA results did not correlate with age (Figure S2A). For patients with the requested TBE diagnostics, the probability of false reactivity in the screening BoDV-1 ELISA slightly increased with age (slope of logistic regression significantly different from 0 with *p* = 0.02). The glomerular filtration rate (GFR) of outpatients after solid organ transplantation did not significantly influence the rate of false ELISA reactivity (Figure S2B).

Evaluating the questionnaire, there was no evidence for an association of already described or putative epidemiological risk factors for BoDV-1 infections such as rural residence, contact to animals, gardening, or direct access from the property to fields/meadows/forests with a false-reactive ELISA screening result (Table 2). Within the cohort of healthy blood donors, an increased odds ratio of 4.85 (95% CI: 1.41–25.86; *p* = 0.01; Pearson’s chi-squared test) for a false-reactive ELISA result was present if the participants had stated in the questionnaire to sometimes walk barefoot outside. In the joint analysis of both healthy blood donors and transplant patients, however, there was no significant association. For the self-reported new onset of neurological symptoms such as headaches, ataxia, paresis, or memory impairment, no association with false-reactive screening results was found (Table S2).

In summary, the lack of association of reactive screening ELISA results with epidemiological or clinical characteristics supports the interpretation as false-reactive ELISA screening results.

### 3.6. Serological Epitope Mapping

Performing a serological linear epitope mapping, we aimed to decipher linear epitopes of antibodies and a possible molecular mimicry as the underlying mechanism for the false-reactive BoDV-1 ELISA screening results.

For this approach, three peptide macroarrays on cellulose membranes were used (Figure S3). Whole BoDV-1 N, X, and P proteins were covered by 15-mer peptides with an overlap of 11 amino acids to neighboring peptides. ELISA and immunoblot positive samples were available for nine BoDV-1 patients. For each patient, one sample (serum/plasma or CSF) was tested. Five sera of patients with a brain biopsy that tested negative for BoDV-1 RNA in RT-qPCR analysis and with a negative serology result (ELISA and iIFA) were included as negative controls. In addition, 9–10 samples from the transplant cohort as well as 3 samples from the blood donor cohort, all false-reactive in the ELISA screening, were tested on the corresponding membrane. Three ELISA negative samples from the blood-donor cohort were included for comparison. For analysis, the base 2 logarithm of background-corrected spot intensities was used. An arbitrary cut-off of 13.5 was established in order to minimize unspecific staining for samples from the negative control.

**Figure 3:**
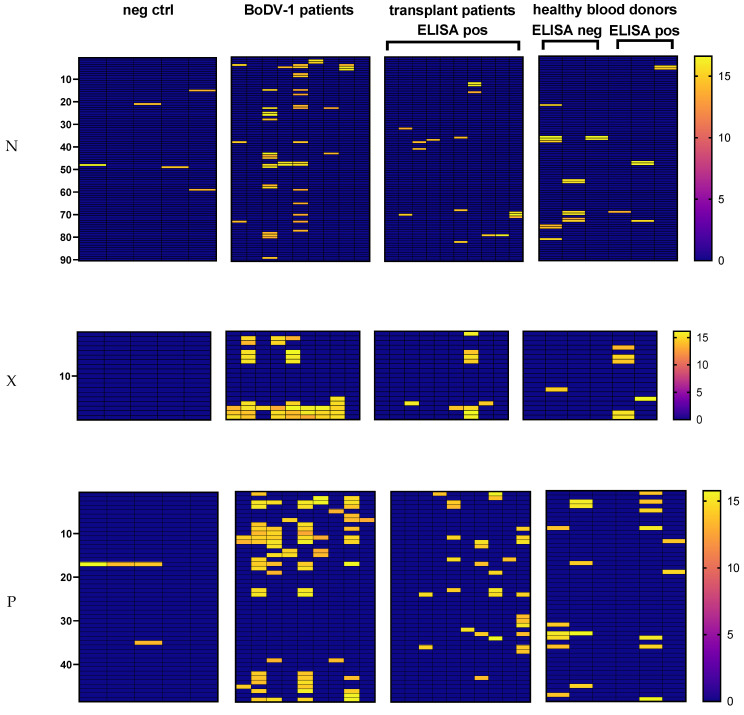
Heatmaps of serological epitope mapping for N (top row), X (middle row), and P proteins (bottom row). The 15-mer peptides are represented in the y-direction from N- (top) to C-terminus (bottom). Individual samples of different cohorts are represented in the x-direction. Values represent the base 2 logarithm-transformed intensity values after background subtraction. An arbitrary cut-off of 13.5 was set to minimize unspecific staining of negative controls samples. Values below the cut-off were set to 0.

The samples of seven out of nine tested patients with confirmed BoDV-1-infection showed spots on the N membrane (number of spots in the range of 2–16) (Figure 3 and Figure S4). One of the two samples that were tested negative by the peptide array was concordantly negative in the anti-N-protein-IgG ELISA, while the other sample tested positive in the ELISA and immunoblot tests. Within the negative control samples, four of five samples showed 1–2 spots on the N peptide array, whereas the samples of all 10 tested patients after solid organ transplantation with a positive anti-1-N-IgG screening ELISA had 1–3 spots. In addition, the samples of all three tested healthy blood donors with a positive ELISA screening were also positive on the array membrane (range 1–3 spots). Unexpectedly, the samples of all three healthy blood donors with negative screening ELISA showed 2–7 spots.

As BoDV-1-specific N peptides, four longer consecutive peptide regions with sequences RRLVDDADAMEDQDLYEPPASLP (amino acids 5–27 of N protein, peptide position 2–4 on membrane), AFVHGGVPRESYLSTPVTRGEQTVVKTAKFYGEKT (amino acids 89–123 of N protein, peptide position 23–28 on membrane), MMAALNRPSHGETATLLQMFNPH (amino acids 169–191 of N protein, peptide position 43–45 on membrane), and AAFYWSKKENPTMAGYRAS (amino acids 305–319 of N protein, peptide position 77–78 on membrane) were identified (Table S3). These united regions were recognized by the samples of six of the seven array-positive BoDV-1 patients with at least two positive samples in each region.

All linear epitopes of samples with false-reactive ELISA results were blasted against a homo sapiens (taxid: 9606) protein database (https://blast.ncbi.nlm.nih.gov (accessed on 10 May 2022)). Additionally, a protein blast was performed independently of the target organisms after exclusion of *Bornaviridae* (taxid: 178830) (Table S4). The linear epitopes were either exclusive for a false-reactive ELISA result or were shared by results correctly identified as positive in the cohort of BoDV-1 patients. In the regions between amino acids (aa) 141 and 163 of N protein (peptides 36–38 on membrane) and between aa 269 and 335 (peptides 68–82 on membrane), the spots of BoDV-1 patients, transplant patients, and healthy blood donors whose sera tested negative or positive in the ELISA overlapped or were found in close proximity. Blasting these regions against homo sapiens (taxid: 9606) resulted in the best alignment with endogenous Bornavirus-like nucleoprotein 2 (E = 0.65) and endogenous Bornavirus-like nucleoprotein 1 (E = 2 × 10^−5^), respectively.

On the X membrane, the samples of all eight patients with confirmed BoDV-1 infection that were reactive in anti-X-IgG ELISA showed spots in a C-terminal region with the peptide sequence PAPEGPQEEPLHDLRPRPA (aa 61–79 of X protein, peptides 16–17 on membrane), which, however, was not exclusively recognized by this cohort (Figure 3 and Figure S4). In addition, the samples of three of these patients had spots in a N-terminal region with the sequence LRLTLLELVRRLNGNATIE (aa 5–23 of X protein, peptides 2–3 on membrane) (Table S3). No spots were found for the sample of the single BoDV-1 patient who tested negative in the anti-X-IgG ELISA screening. Over all samples, spots in the N- and C-terminal region on the X membrane were indicative of ELISA reactivity as ELISA negative sera showed no spots in both of these regions. With regard to samples with a false-reactive ELISA result, 50% did not stain any spot on the X membrane, a third showed a single spot, and one sample of the transplant outpatients and one of the healthy blood donors had seven and four spots, respectively. Most spots of the two samples with multiple spots were located in the N- and C-terminal regions where BoDV-1 patients also shared epitopes. Two peptides that were exclusively attributable to a false-reactive ELISA were located in the N-terminal region. A blast against a protein database showed the lowest E values for alignments either with a human or a bacterial protein (Table S4).

On the P membrane, all ELISA positive BoDV-1 samples had positive spots (range 1–18) (Figure 3 and Figure S4). The N-terminal peptide region LRRERPGSPRPRKVPRNALTQPV (aa 21–43, peptide positions 6–8 on P membrane) (recognized by 4 patients) and the C-terminal peptide PPRIYPQLPSAPTTD (aa 181–195 of P protein, peptide position 46 on membrane) (recognized by 3 patients) were specific for BoDV-1 (Table S3). Samples of negative control had a maximum of two spots, and ELISA negative samples from healthy blood donors had a maximum of six spots. Regarding samples with false-reactive ELISA, three out of thirteen tested samples had no corresponding spot on the P membrane, three samples stained one spot, while seven out of thirteen samples had more than two spots (maximum of ten spots). While only a few peptides were exclusively indicative for a false-reactive ELISA result, most samples with a false-reactive ELISA overlapped with epitope regions of BoDV-1-infected patients (Table S4).

In the region ALQVETIQTAQRCDHSDSIRILGENIKILDRSMKTMMETMKLMMEKV (aa 113–155 of P protein, peptide positions 29–37 on membrane), however, only spots from samples with either a negative ELISA or a false-reactive ELISA were found, whereas the BoDV-1 samples did not stain any spots in this region. When the whole peptide sequence was blasted against a homo sapiens protein database (taxid: 9606), only possible alignments with comparatively high E values were found (E = 2.6 for alternative protein RBM28, E = 2.9 for protein CLEC16A). After the exclusion of *Bornaviridae* (taxid: 178830), the sequence was blasted organism-independently. Best matches were found for proteins of the snake *bitis gabonica* (E = 0.019) and for bacteria of the phylum *Zixibacteria* (E = 0.14), the family *Acholeplasmatacea* (E = 0.20), the class *Elusimicrobia* (E = 0.70), the species *Cryomorpha ignava* (E = 0.92), and the phylum *Firmicutes* (E = 1.1). As *bitis gabonica* is not endemic in Germany, cross-reactivity with antibodies against environmental bacteria or bacterial commensals could explain the spots on the P membrane in this region.

**Figure 4:**
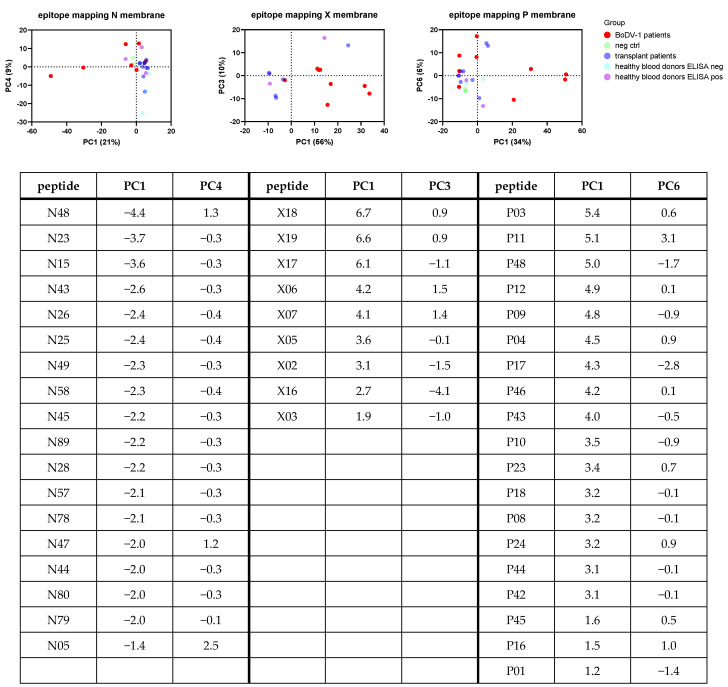
Principal component analysis (PCA) of serological epitope mapping. All 15-mer peptides of N, X, and P proteins were included as individual variables. A PCA test was performed for each membrane separately. Dots represent individual samples of the cohorts of negative controls (green), BoDV-1 patients (red), transplant patients with positive ELISA (blue), healthy blood donors with positive ELISA (purple), and healthy blood donors with negative ELISA (turquoise). Patients with BoDV-1 infection were best separated by principal component 1 (PC1) and PC4 (N membrane), by PC1 and PC3 (X membrane) and by PC1 and PC6 (P membrane). Percentages in parentheses provide the contribution to total variance for each PC. The most important loadings of PC in order to separate patients with BoDV-1 infection are given below.

As an overall analysis, a principal component analysis (PCA) was performed for the samples tested on each membrane (Figure 4). Samples belonging to patients with BoDV-1 infection were easiest to cluster based on the PC1 and PC3 of the X membrane analysis, whereas only four patients with BoDV-1 infection clustered together based on the analysis of the N and P membrane. Loadings of PC contributing most to separation helped to identify peptides or the combination of peptides indicating ELISA results correctly identified as positive. Samples with false-reactive ELISA results did not uniformly cluster.

## 4. Discussion

The aim of the BoSOT study was to assess whether there are oligo- or asymptomatic BoDV-1 infections in healthy blood donors or patients after solid organ transplantation in a well-known endemic area with previous human and animal cases [18,21]. In comparison, patients with requested TBE diagnostics were tested for a BoDV-1 infection.

In a cohort of 216 healthy blood donors as well as in a cohort of 280 transplant patients with solid organ transplantation, no iIFA-confirmed positive serological results were found. Screening of transplant patients was of major interest as the first cases of confirmed human BoDV-1 encephalitis were described in the context of a solid organ transplantation with an undiagnosed organ donor transmitting the virus via post mortem kidney and liver transplantation to three recipients [2]. In our cohort of transplant recipients in a comparatively stable phase of median eight years after solid organ transplantation, we did not find a confirmed positive case based on iIFA data and thus no increased serological risk signal in comparison to healthy blood donors. The current study, however, may not be sufficiently powered to address the question whether solid organ transplantation is associated with an increased risk for rare symptomatic BoDV-1 infections manifesting as encephalitis.

One new case of a human BoDV-1 infection, however, was discovered within a cohort of 288 patients for whom TBE diagnostics were requested from serum/plasma and/or CSF samples between May 2021 and July 2022. Assuming a diagnosis of encephalitis for 90% (258) of these patients, for whom a corresponding CSF sample was collected, leads to a fraction of 0.39% BoDV-1 cases in relation to cases of suspected encephalitis. It cannot be excluded that the remaining 10% of individuals of this cohort with only serum or plasma samples available may rather represent a collective of patients after tick bite without severe neurological disease or of individuals for whom it was intended to evaluate the immunogenicity of a TBE vaccination. Within the period of 14 months, 17 cases of TBE encephalitis were diagnosed, where 15 cases were based on an elevated CSF IgG index and an additional 2 patients under severe immunosuppression were based on a positive TBE virus RT-qPCR from CSF, and which resulted in a fraction of 6.59% of TBE cases in relation to the cases of suspected encephalitis.

In summary, an iIFA-confirmed BoDV-1 positive serological result from a serum/plasma sample screening as well as a positive RT-qPCR result from a CSF sample screening was only found in the cohort of patients with neurological symptoms from whom a CSF sample was collected. The current study found no clear evidence for oligo- or asymptomatic BoDV-1 infections and thus supports the notion that the primary manifestation of BoDV-1 infection is severe encephalitis, even if the study may not be sufficiently powered to exclude any BoDV-1-associated oligo- or asymptomatic cases. In particular, solid organ transplantation and subsequent immunosuppression were not associated with a proven seropositivity for anti-BoDV-1-IgG based on an iIFA as confirmatory test, whereas the specificity of the screening ELISA was limited in this cohort.

Our finding of a very low seroprevalence is in line with another serological screening study published in 2019 in which 1,109 participants (healthy blood donors and veterinarians) living in endemic regions in southern Germany were examined [23]. Only one serum sample from a veterinarian was tested positive by iIFA testing. According to the questionnaire, the person suffered from joint pain. The further fate of the person could not be followed due the anonymized design of the study. The measured high avidity of the antibodies is, however, indicative for a chronic course of the disease. Thus, oligo- or asymptomatic courses of BoDV-1 infections cannot be totally excluded but are likely to be very rare as well. Our current study supports the conclusion that the ratio of oligo-/asymptomatic to symptomatic cases with severe encephalitis is small if not zero. As data of long-time follow-up serology in patients suffering from BoDV-1 are lacking, the impact of waning antibodies on very low seroprevalence, however, cannot be sufficiently evaluated.

As a side result of the BoSOT study, the performances of different serological methods were compared. The specificity of the screening ELISA was limited when based on single antigen positivity. Interestingly, specificity was different between the cohorts, with most restrictions for the cohort of transplant patients. In our previous publication on the BoDV-1 ELISA, we proposed dual positivity for a more trustworthy serological result [21]. This criterion increased the specificity of the ELISA to 97.7% over all cohorts in this study. However, this dual-positivity criterion will affect the sensitivity. While in our previous publication, all patients developed positivity against at least two antigens during the time course of the disease, the only available sample of the newly BoDV-1-diagnosed patient in this study was only the positive result in the anti-P-IgG. Similarly, the titer measured in the iIFA test was relatively low (1:40). Of note, the patient received immunosuppressive treatment at the time of sampling, possibly contributing to the low antibody titer.

In conclusion, serological tests based on a single recombinant BoDV-1 antigen alone are not suitable for BoDV-1 diagnostics. An algorithm combining tests based on different BoDV-1 antigens and re-testing of all reactive results by a confirmatory iIFA is necessary. As immunoblots using the same recombinant viral proteins had a good correlation with ELISA results, the cause of restricted specificity is most likely due to the viral recombinant proteins themselves. In order to elucidate possible mechanisms, we performed a serological linear epitope mapping.

As a first result, we identified peptides or peptide regions within the N, X, and P protein to be specific for IgG responses of confirmed BoDV-1 patients.

Interestingly, one of our N protein-derived peptide regions that was found to be specific for BoDV-1 patients was previously identified as the epitope of the anti-BoDV-1-N-protein monoclonal mouse antibody Bo18, for which the linear epitope LYEPPASLP (amino acids 19–27 of N protein) was published [24,25].

In the same study, the peptide QLPSAPTADEWD (amino acids 187–198 of P protein) was found as a linear epitope of the monoclonal anti-BoDV-1-P-protein antibody P21E7. In our epitope mapping, this peptide was recognized by samples of four patients with confirmed BoDV-1 infection as well as by two samples of healthy blood donors (one negative in ELISA, one positive in ELISA).

Evaluating our epitope mapping with regard to a general underlying mechanism for false-reactive ELISA results, we did not find an overall explanation. Samples with false-reactive ELISA showed one of the following possible results in the linear epitope mapping: First, some of the samples did not stain any spots. For four samples with this characteristic that were reactive in the anti-X-IgG-ELISA, a GST-tag-directed binding of antibodies was excluded (Figure S5). Second, some samples recognized peptide epitopes that were not shared by samples of BoDV-1-infected patients. For those peptides, a protein BLAST search was performed. Within a region of the P protein that was only recognized by ELISA negative or ELISA false-reactive samples but not by samples of BoDV-1 patients, alignments with proteins of certain bacteria were likelier than with human proteins. Thus, for some false-reactive ELISA results, cross-reactivity with antibodies against bacteria could be an explanation. Third, some epitopes of samples with false-reactive ELISA results were shared with epitopes of confirmed BoDV-1 patients. When we performed a BLAST search for two regions containing multiple epitopes within the N protein, where samples of BoDV-1 patients as well as samples of participants with negative or false-reactive ELISA results recognized peptides, we found an alignment of these regions with endogenous Bornavirus-like elements (EBLN). A homologous sequence of BoDV-1 N protein within the human genome was first described in 2010 when a BLAST search was performed in a study that was intended to elucidate mechanisms for the persistence of BoDV-1 infections [26]. Since then, several homologous sequences of BoDV-1 N protein in the human genome and in the genome of a wide array of species were found and were termed as endogenous Bornavirus-like elements (EBLN). The tissue-dependent transcription of some of these genes as well as the translation of at least the ORF-encoding human EBLN hsEBLN-2 into a functional mitochondrial protein with certain functions was demonstrated [27,28,29]. In addition, homologous sequences for BoDV-1 M, G, and L protein have been found in the genome (termed EBLM, EBLG and EBLL) [27]. There are several possible explanations as to why no integrations of X or P protein have been found so far. BoDV-1 *X/P* genes tend to be more mutable than other BoDV-1 genes. Thus, the viral evolution after the integration in the genome could have led to so many differences that a BLAST search is not able to find alignments [27]. Another explanation could be that P protein in contrast to N protein has been described in animal studies as a pathogenic factor whose overexpression in cells could be associated with adverse effects on host animals [27]. Autoantibodies to the hsEBLN-2-encoded mitochondrial protein could be an explanation why the aligned region of BoDV-1 N protein is recognized by several samples in our epitope mapping; why only some of these autoantibodies lead to positive ELISA results, however, remains elusive. As sequences homologous with X/P protein have not been found in the human genome so far, this mechanism does not readily explain the increased false reactivity rates in anti-P-IgG ELISA, especially within the cohort of transplant patients. The absence of a clear association of epidemiological or clinical risk factors underlines the interpretation as false-reactive results of our ELISA screening test. The iIFA test performed the best in terms of specificity; an explanation could be the 3D structure of the RNP complex where viral proteins are assembled with viral RNA within the cell nucleus.

## 5. Conclusions

In conclusion, we diagnosed a new BoDV-1 infection in a patient with severe neurological symptoms but did not find any serological evidence for oligo- or asymptomatic BoDV-1 infections in an endemic region. In patients with a new onset of severe neurological symptoms who live in or visited a region well-known to be endemic for BoDV-1, BoDV-1 diagnostics should be initiated even when the cell count in the CSF is normal. According to a recently published case definition, a confirmed BoDV-1 diagnosis requires the detection of BoDV-1-specific RNA or proteins [19]. As viral RNA loads in CSF are relatively low, an RT-qPCR test from this material has only limited sensitivity, sometimes leading to the requirement of a brain biopsy or a post mortem tissue section to fulfil the definition of a confirmed case. Therefore, serology should always be performed in parallel. Serological tests that are based on recombinant antigens should always be interpreted in combination with an iIFA test, as we identified cross-reactivities with human or bacterial proteins as potential underlying mechanisms for limited specificity.

## Figures and Tables

**Table 1:** Characteristics of study cohorts.

	Healthy Blood Donors	Outpatients after Solid Organ Transplantation	Patients with Requested TBE Diagnostics
*N*	216	280	Serum/Plasma: 288 CSF: 258
Inclusion period (start and end date)	2 November 2021 18 May 2022	21 May 2021 12 January 2022	10 May 2021 28 July 2022
Age [years]	Median: 28 (IQR: 25–37)	Median: 60 (IQR: 52–67)	Median: 57 (IQR: 40–66)
Sex [no.]	Female: 74 (34%) Male: 142 (66%)	Female: 100 (36%) Male: 180 (64%)	Female: 134 (47%) Male: 154 (53%)
BMI [kg/m^2^]	Median: 24.6 (range: 19.0–46.6)	Median: 26.6 (range: 17.8–46.4)	NA
Solid organ transplantation	No: 193 (89%) NA: 23 (11%)	Kidney: 254 (91%) Pancreas: 2 (<1%) Liver: 1 (<1%) Kidney + pancreas: 19 (7%) Kidney + heart: 2 (<1%) NA: 2 (<1%)	NA
Interval: solid organ transplantation to sample collection [years]	NA	Median: 8.0 (IQR: 4.0–12.1)	NA
Transplant rejection [no.]	NA	No: 114 (41%) Yes: 76 (27%) Unknown: 89 (32%) NA: 1 (<1%)	NA
GFR (CKD-EPI) [ml/min/1.73 m^2^]	NA	Median: 48 (range: 6–109)	NA

(NA = not available).

**Table 2:** Contingency analysis of questionnaire and ELISA screening (reactivity against ≥ 1 antigen).

	Healthy Blood Donors	Outpatients After Solid Organ Transplantation	Patients with Requested TBE Diagnostics
Sex: female	OR: 1.01 95% CI: 0.41–2.35 *p* = 0.99	OR: 1.59 95% CI: 0.91–2.76 *p* = 0.08	OR: 0.87 95% CI: 0.41–1.81 *p* = 0.69
Transplanted organ	NA	*p* = 0.30 (FT)	NA
Organ rejection	NA	OR: 1.08 95% CI: 0.56–2.06 0.80	NA
Contact to animals	OR: 1.11 95% CI: 0.49–2.57 *p* = 0.79	OR: 1.00 95% CI: 0.58–1.72 *p* = 1.00	NA
Residence in village <10,000 inhabitants	OR: 1.03 95% CI: 0.37–2.59 *p* = 0.95	OR: 0.63 95% CI: 0.35–1.12 *p* = 0.09	NA
Practicing gardening	OR: 0.58 95% CI: 0.24–1.35 *p* = 0.17	OR: 0.71 95% CI: 0.41–1.24 *p* = 0.20	NA
Stone wall in garden	OR: 0.79 95% CI: 0.29–1.96 *p* = 0.58	OR: 0.97 95% CI: 0.54–1.74 *p* = 0.92	NA
Contact to shrews	OR: 0.77 95% CI: 0.18–2.46 *p* = 0.79 (FT)	OR: 1.14 95% CI: 0.58–2.17 *p* = 0.68	NA
Walking barefoot outside	OR: 4.85 95% CI: 1.41–25.86 *p* = 0.01	OR: 0.95 95% CI: 0.52–1.72 *p* = 0.86	NA
Residence: countryside	OR: 0.48 95% CI: 0.15–1.29 *p* = 0.12	OR: 0.90 95% CI: 0.51–1.61 *p* = 0.70	NA
Access to fields, meadows, forests	OR: 0.96 95% CI: 0.42–2.19 *p* = 0.92	OR: 1.11 95% CI: 0.62–2.00 *p* = 0.71	NA

Statistical analysis with Pearson’s chi-squared test (or Fisher’s exact test (FT) where indicated). (OR = odds ratio, 95% CI = 95% confidence interval of OR).

## Data Availability

The data presented in this study are available on request from the corresponding author.

## Supplementary Materials

The following supporting information can be downloaded at: https://www.mdpi.com/article/10.3390/v15010188/s1: Figure S1 (Reproducibility of reactive BoDV-1 screening ELISA), Figure S2 (Logistic regression age/GFR and reactive BoDV-1 screening ELISA), Figure S3 (Linear epitope mapping with N, X and P membranes), Figure S4 (Analysis of epitope mapping membranes by number of positive samples), Figure S5 (Immunoblots BoDV-1 X protein), Table S1 (Questionnaire with epidemiological risk factors), Table S2 (Contingency analysis of clinical signs (questionnaire) and reactive ELISA screening (reactivity against ≥ 1 antigen)), Table S3 (Peptides specific for patients with BoDV-1 infection), Table S4 (Peptides associated with false-reactive ELISA results).
