# Supplementary material for: Human Infections with Borna Disease Virus 1 (BoDV-1) Primarily Lead to Severe Encephalitis: Further Evidence from the Seroepidemiological BoSOT Study in an Endemic Region in Southern Germany
Source: Viruses. 2023 Jan 9;15(1):188. doi: 10.3390/v15010188 (PMC9867173; doi:10.3390/v15010188)
Supplement: Supplementary file 1 [file viruses-15-00188-s001.zip › Bauswein et al._BoDV-1 seroepidemiology_supplementary figures.pptx]

## Slide 1
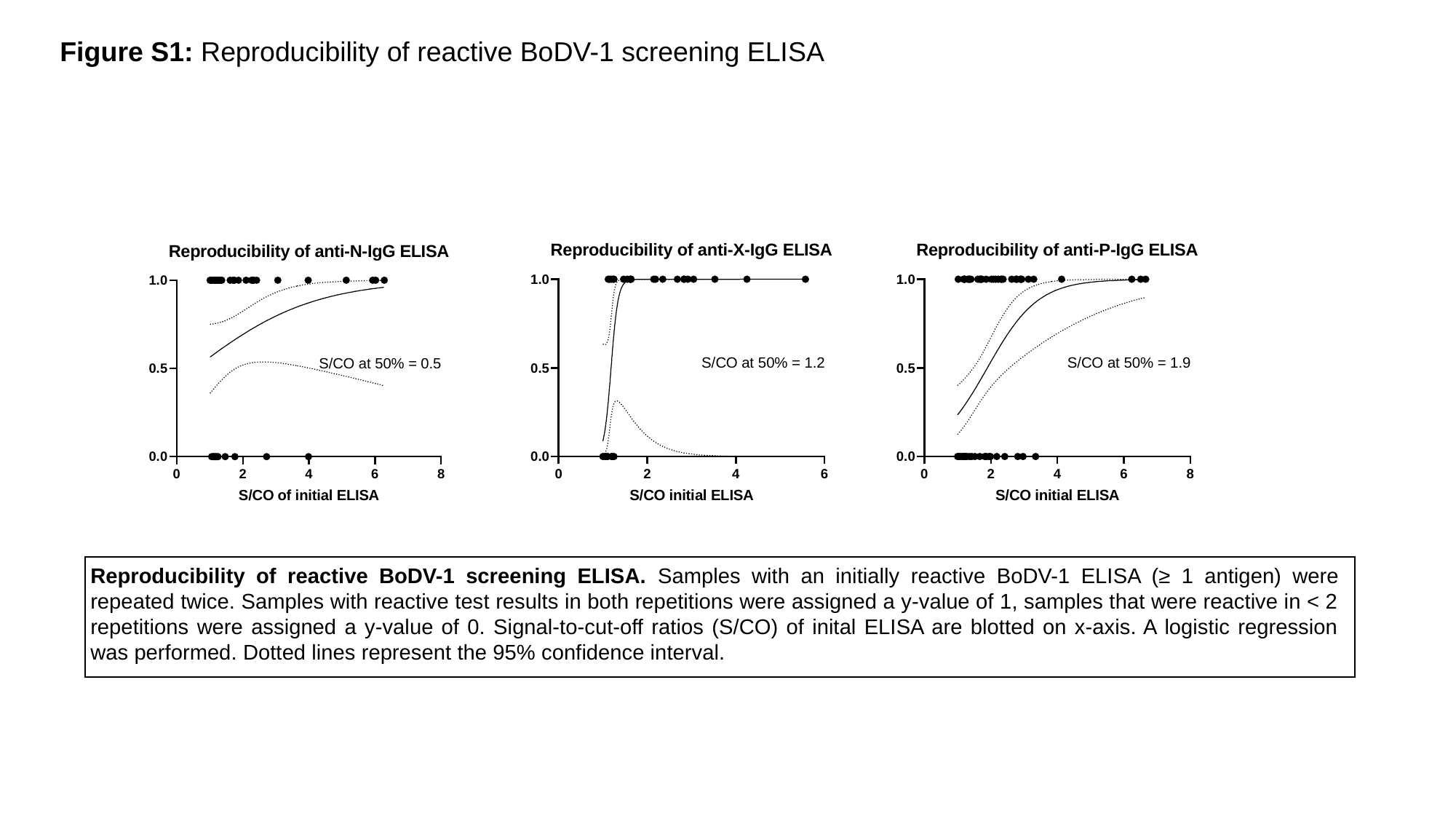

Figure S1: Reproducibility of reactive BoDV-1 screening ELISA
Reproducibility of reactive BoDV-1 screening ELISA. Samples with an initially reactive BoDV-1 ELISA (≥ 1 antigen) were repeated twice. Samples with reactive test results in both repetitions were assigned a y-value of 1, samples that were reactive in < 2 repetitions were assigned a y-value of 0. Signal-to-cut-off ratios (S/CO) of inital ELISA are blotted on x-axis. A logistic regression was performed. Dotted lines represent the 95% confidence interval.

## Slide 2
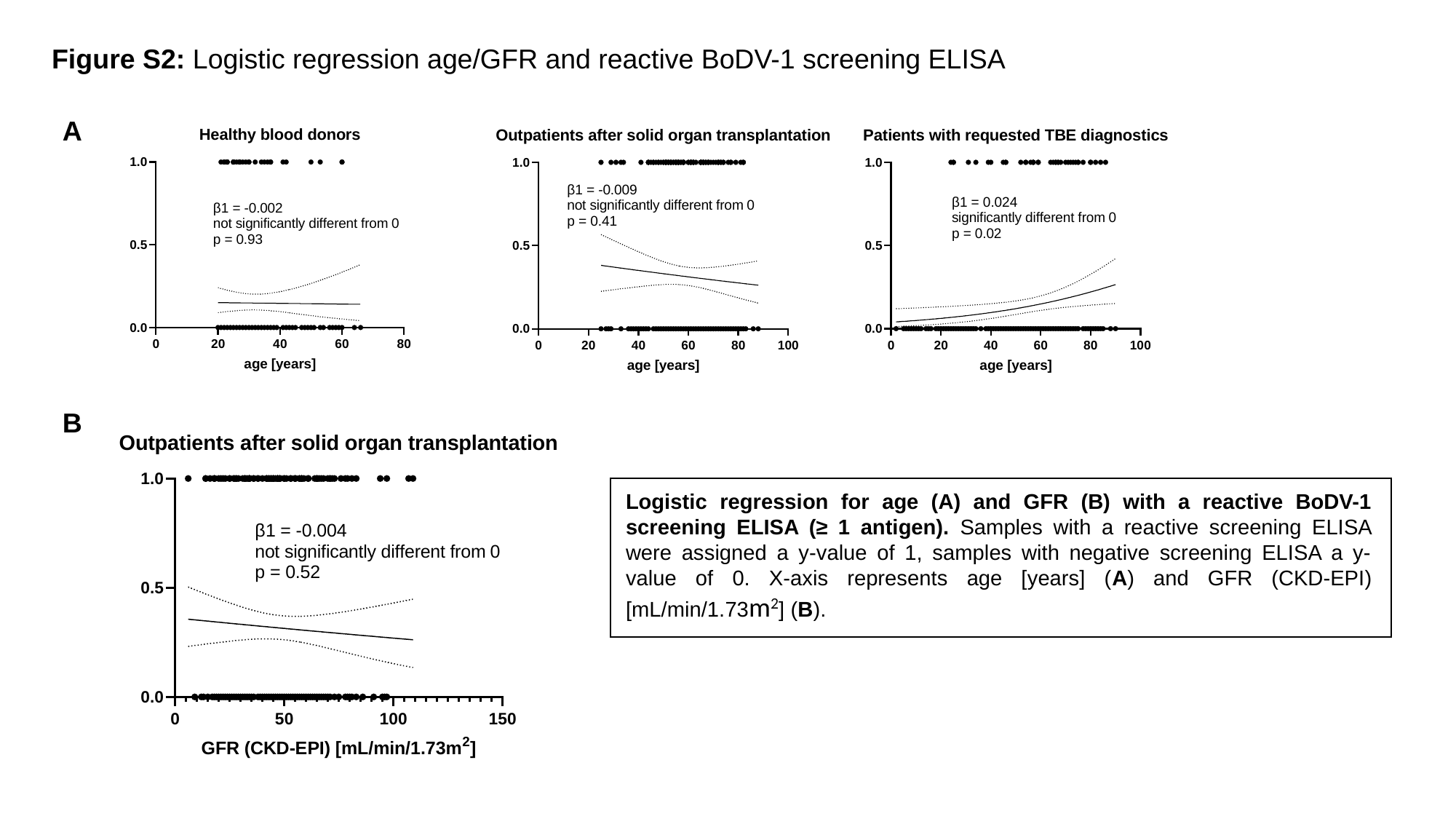

Figure S2: Logistic regression age/GFR and reactive BoDV-1 screening ELISA
A
B
Logistic regression for age (A) and GFR (B) with a reactive BoDV-1 screening ELISA (≥ 1 antigen). Samples with a reactive screening ELISA were assigned a y-value of 1, samples with negative screening ELISA a y-value of 0. X-axis represents age [years] (A) and GFR (CKD-EPI) [mL/min/1.73m2] (B).

## Slide 3
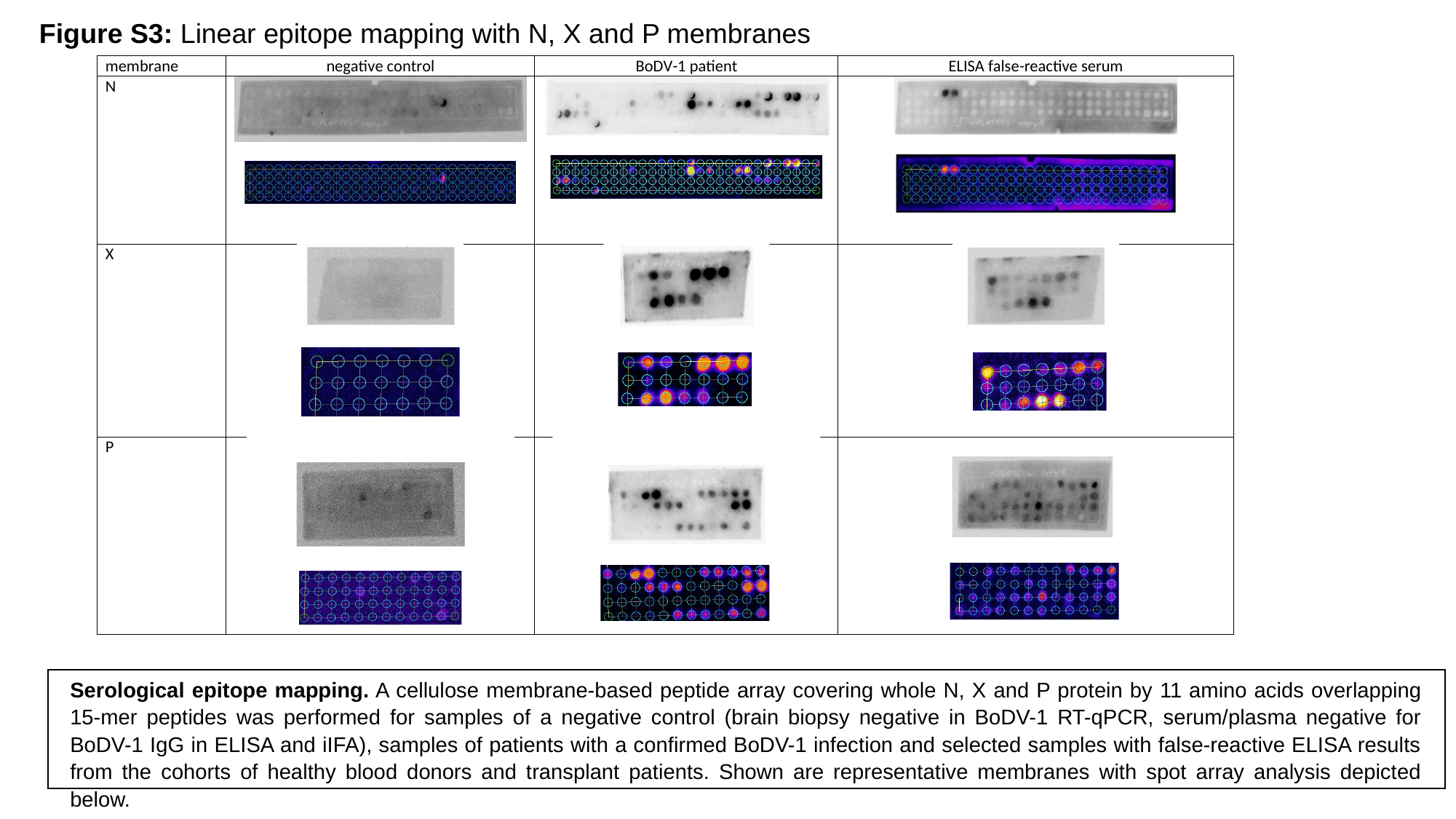

Figure S3: Linear epitope mapping with N, X and P membranes
Serological epitope mapping. A cellulose membrane-based peptide array covering whole N, X and P protein by 11 amino acids overlapping 15-mer peptides was performed for samples of a negative control (brain biopsy negative in BoDV-1 RT-qPCR, serum/plasma negative for BoDV-1 IgG in ELISA and iIFA), samples of patients with a confirmed BoDV-1 infection and selected samples with false-reactive ELISA results from the cohorts of healthy blood donors and transplant patients. Shown are representative membranes with spot array analysis depicted below.

## Slide 4
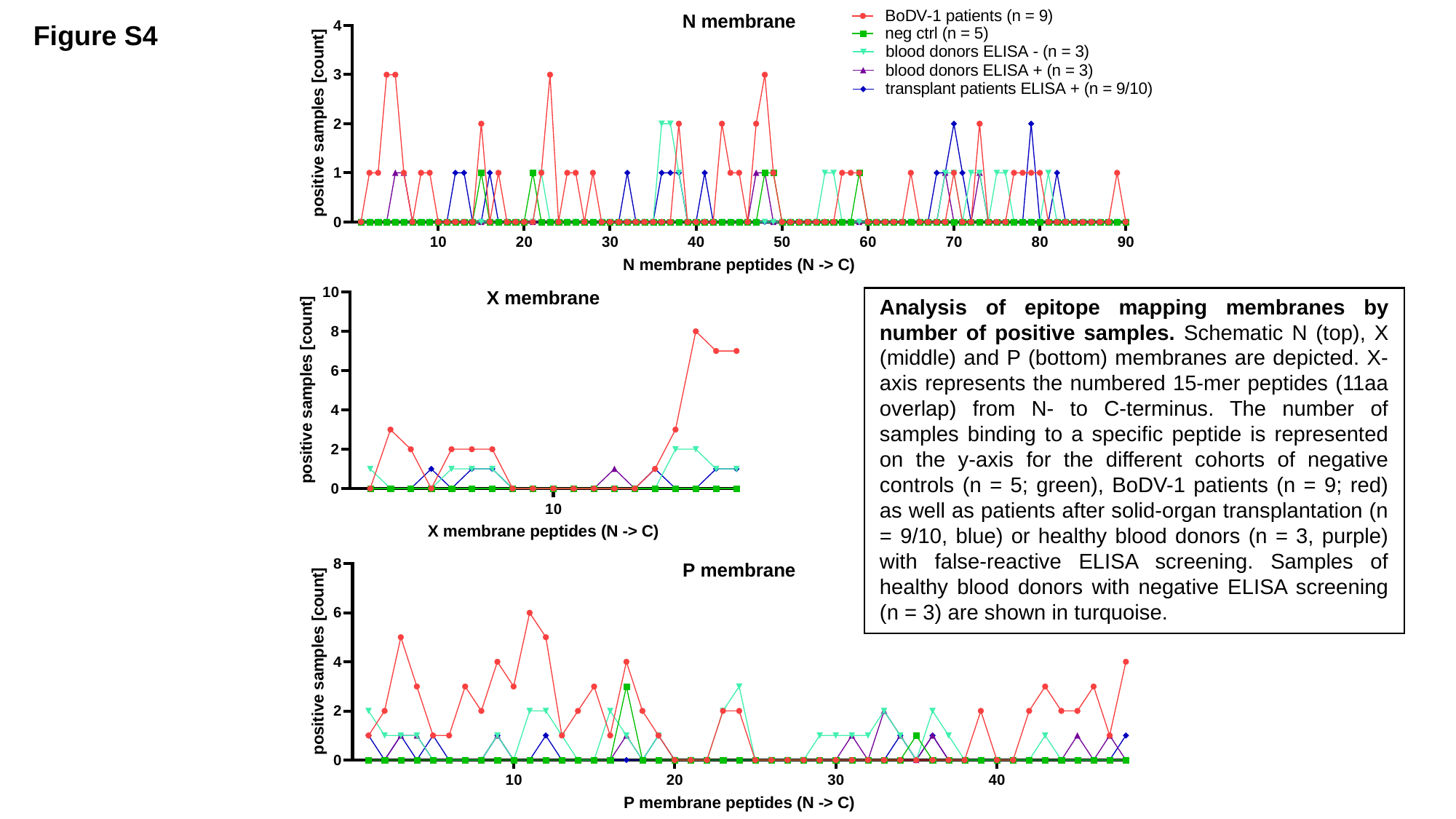

Figure S4
Analysis of epitope mapping membranes by number of positive samples. Schematic N (top), X (middle) and P (bottom) membranes are depicted. X-axis represents the numbered 15-mer peptides (11aa overlap) from N- to C-terminus. The number of samples binding to a specific peptide is represented on the y-axis for the different cohorts of negative controls (n = 5; green), BoDV-1 patients (n = 9; red) as well as patients after solid-organ transplantation (n = 9/10, blue) or healthy blood donors (n = 3, purple) with false-reactive ELISA screening. Samples of healthy blood donors with negative ELISA screening (n = 3) are shown in turquoise.

## Slide 5
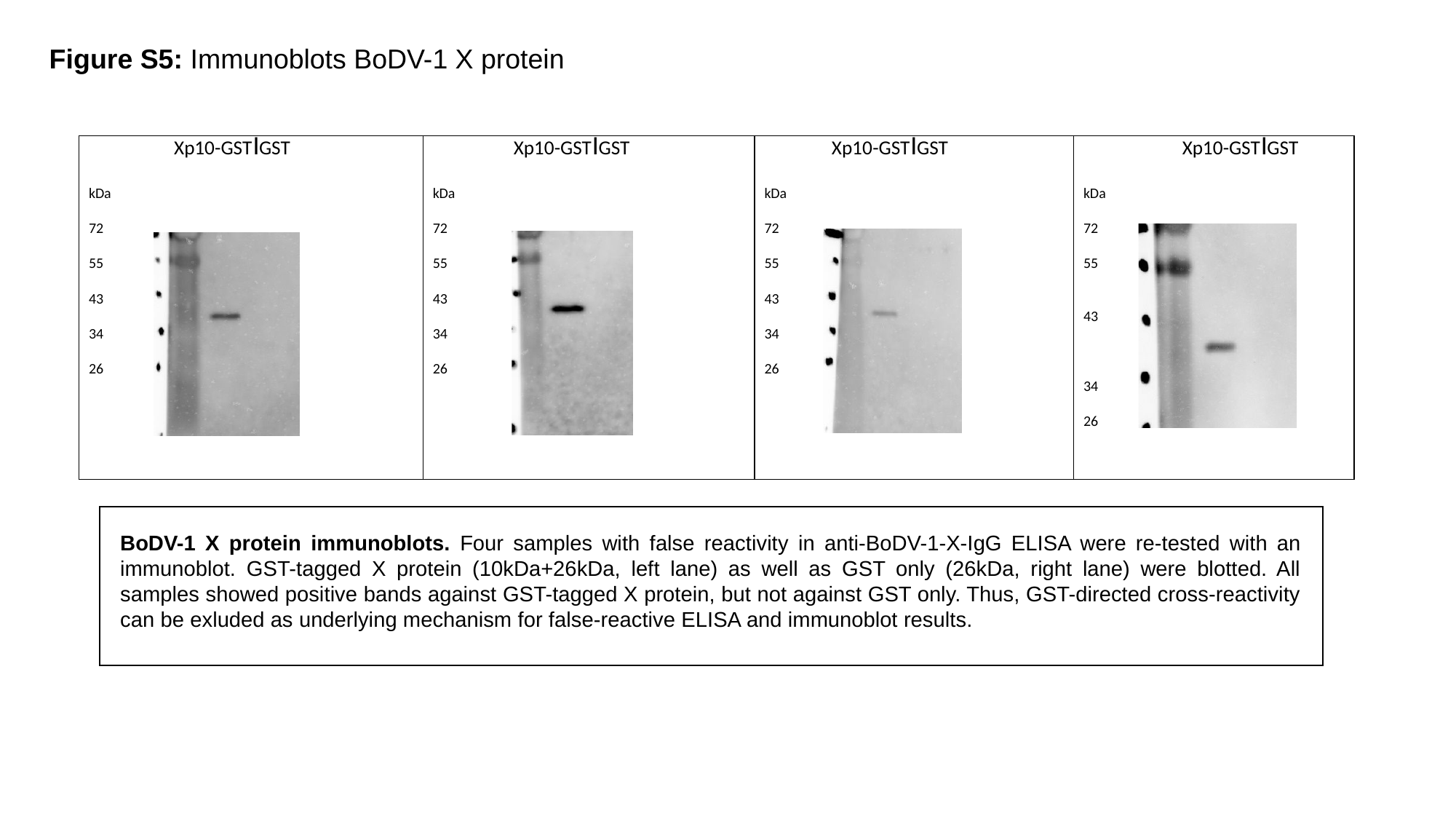

Figure S5: Immunoblots BoDV-1 X protein
BoDV-1 X protein immunoblots. Four samples with false reactivity in anti-BoDV-1-X-IgG ELISA were re-tested with an immunoblot. GST-tagged X protein (10kDa+26kDa, left lane) as well as GST only (26kDa, right lane) were blotted. All samples showed positive bands against GST-tagged X protein, but not against GST only. Thus, GST-directed cross-reactivity can be exluded as underlying mechanism for false-reactive ELISA and immunoblot results.
