# Supplementary material for: Human Infections with Borna Disease Virus 1 (BoDV-1) Primarily Lead to Severe Encephalitis: Further Evidence from the Seroepidemiological BoSOT Study in an Endemic Region in Southern Germany
Source: Viruses. 2023 Jan 9;15(1):188. doi: 10.3390/v15010188 (PMC9867173; doi:10.3390/v15010188)
Supplement: Supplementary file 1 [file viruses-15-00188-s001.zip › Bauswein et al._BoDV-1 seroepidemiology_supplementary tables.pdf]

Table S1: Questionnaire with epidemiological risk factors

	healthy blood donors (n = 216)	outpatients after solid-organ transplantation (n = 280)
residence	city/town: 141 (65%)<br>countryside: 67 (31%)<br>ambiguous answer: 4 (2%)<br>NA: 4 (2%)	city/town: 88 (31%)<br>countryside: 186 (66%)<br>ambiguous answer: 1 (< 1%)<br>NA: 4 (2%)
residence in village < 10.000 inhabitants	no: 160 (74%)<br>yes: 52 (24%)<br>NA: 4 (2%)	no: 93 (33%)<br>yes: 177 (63%)<br>ambiguous answer: 1 (< 1%)<br>NA: 9 (3%)
property with direct access to fields, meadows or forests	no: 117 (54%)<br>yes: 94 (44%)<br>NA: 5 (2%)	no: 91 (33%)<br>yes: 185 (66%)<br>ambiguous answer: 2 (< 1%)<br>NA: 2 (< 1%)
practicing gardening	no: 114 (53%)<br>yes: 95 (44%)<br>ambiguous answer: 1 (< 1%)<br>NA: 4 (2%)	no: 98 (35%)<br>Yes: 178 (64%)<br>ambiguous answer: 1 (< 1%)<br>NA: 3 (1%)
garden with stone wall	no: 149 (69%)<br>yes: 61 (28%)<br>NA: 6 (3%)	no: 174 (62%)<br>yes: 92 (33%)<br>NA: 14 (5%)
animal contact	no: 96 (44%)<br>yes: 113 (52%)<br>NA: 5 (2%)	no: 132 (47%)<br>yes: 145 (52%)<br>NA: 2 (< 1%)
contact to cats	yes: 66 (31%)	yes: 61 (22%)
contact to shrews	no: 179 (83%)<br>yes: 32 (15%)<br>NA: 4 (2%)	no: 210 (75%)<br>yes: 61 (22%)<br>NA: 9 (3%)
walking barefoot outdoors	no: 148 (69%)<br>yes: 63 (29%)<br>NA: 4 (2%)	no: 195 (70%)<br>yes: 80 (29%)<br>NA: 5 (2%)

Table S2: Contingency analysis of clinical signs (questionnaire) and reactive ELISA screening (reactivity against ≥ 1 antigen)

new onset/aggravation of	healthy blood donors	outpatients after solid-organ transplantation
headache	OR not defined/not calculated<br><br>p = 1.00	OR: 2.18<br>95% CI: 0.79-5.91<br>p = 0.09
short-time memory impairment	OR: 1.67<br>95% CI: 0.16-9.36<br>p = 0.63	OR: 0.75<br>95% CI: 0.20-2.30<br>p = 0.80
long-time memory impairment	OR not defined/not calculated<br><br>p = 1.00	OR: 1.59<br>95% CI: 0.57-4.22<br>p = 0.34
visual impairment	OR not defined/not calculated<br><br>p = 0.36	OR: 1.54<br>95% CI: 0.76-3.09<br>p = 0.23
smelling/tasting impairment	OR not defined/not calculated<br><br>p = 1.00	OR: 1.47<br>95% CI: 0.42-4.81<br>p = 0.57
behavioral/personality change	OR not defined/not calculated<br><br>p = 1.00	OR: 2.15<br>95% CI: 0.39-11.83<br>p = 0.28
frequent falls	not defined/not calculated	OR: 1.31<br>95% CI: 0.27-5.32<br>p = 0.74
gait ataxia	OR not defined/not calculated<br><br>p = 1.00	OR: 0.76<br>95% CI: 0.21-2.34<br>p = 0.80
numbness of extremities	OR not defined/not calculated<br><br>p = 1.00	OR: 1.44<br>95% CI: 0.62-3.24<br>p = 0.33
unable to walk long distance	OR not defined/not calculated<br><br>p = 0.15	OR: 1.09<br>95% CI: 0.52-2.20<br>p = 0.86
paresis	OR not defined/not calculated<br><br>p = 1.00	OR: 1.43<br>95% CI: 0.66-3.03<br>p = 0.35

statistical analysis with Fisher´s exact test

(OR = odds ratio, 95% CI = 95% confidence interval of OR)

Table S3: Peptides specific for patients with BoDV-1 infection

protein	peptide position on membrane	amino acids of protein	peptide sequence	no. of positive samples	homology, human proteins (taxid: 9606)	E value
N	2-4	5-27	RRLVDDADAMEDQDLYEPPASLP	4	leptin receptor overlapping transcript like 1	0.058
N	23-28	89-123	AFVHGGVPRESYLSTPVTRGEQTVVKTAKFYGEKT	3	leucine rich repeat containing 5 variant	5.7
N	43-45	169-191	MMAALNRPSHGETATLLQMFNPH	2	endogenous Bornavirus like nucleoprotein 2	0.084
N	57-58	225-239	QIKLVASYAQMTTYTTIKE	1	endogenous Bornavirus like nucleoprotein 1	0.13
N	65	257-271	VVAYEIRDELEVSAK	1	ribosomal protein S6 kinase alpha-5	4.6
N	77-78	305-319	AAFYWSKKENPTMAGYRAS	2	olfactory receptor 4B1	3.0
N	89	353-367	LSGEISAIMKMIGVT	1	fibrous sheath-interacting protein 2	9.3
X	2-3	5-23	LRLTLLELVRRLNGNATIE	3	immunoglobulin heavy chain junction region	7.8
P	6-8	21-43	LRRERPGSPRPRKVPRNALTQPV	4	Ras association domain family member 5<br>PDCD7 protein<br>DNA-binding protein A<br>HYA22<br>zinc finger protein 335	0.06<br>0.17<br>0.33<br>0.47<br>0.93
P	18	69-83	LSNDELIKKLVTELA	2	zinc finger protein 469	0.009
P	39	153-167	KLMMEKVDLLYASTA	2	hCG2036668	9.2
P	42	165-179	STAVGTSAPMLPSHP	2	mediator complex subunit 13L	9.3
P	44	173-187	PMLPSHPAPPRIYPQ	2	alternative protein KLC2	0.4
P	46	181-195	PPRIYPQLPSAPTTD	3	immunoglobulin heavy chain junction region	2.8

Peptides that were found to be specific for samples of patients with BoDV-1 infection in a linear epitope mapping were blasted against a human protein database (https://blast.ncbi.nlm.nih.gov). All results with E values < 1 are given. For peptides with insignificant alignments (E value > 1), the lowest E value is shown.

Table S4: Peptides associated with false-reactive ELISA results

protein	peptide position on membrane	amino acids of protein	peptide sequence	cohort	no. of positive samples	homology, human proteins (taxid: 9606)	E value	homology, organism-independent, exclusion of *Bornaviridae* (taxid: 178830)	E value
N	5-6	17-35	QDLYEPPASLPKLPGKFLQ	blood donor (BoDV-1)	1 (4)	4-hydroxy-phenylpyruvate-dioxygenase	2.1	CehA/McbA family metallohydrolase [Deltaproteobacteria bacterium]	8.8
N	12-13	45-63	PGIGHEKDIRQNAVALLDQ	transplant	1	G protein subunit beta 2	1.5	endogenous Bornavirus-like nucleoprotein 2 [Ictidomys tridecemlineatus]	0.78
N	32	125-139	QRDLTELEISSIFSH	transplant	1	calcium/calmodulin-dependent 3',5'-cyclic nucleotide phosphodiesterase 1C	9.2	pseudouridine synthase [unclassified Clostridium]<br><br>endogenous Bornavirus-like nucleoprotein 2 [Ictidomys tridecemlineatus]	0.14<br><br>0.56
N	36-38	141-163	CSLLIGVVIGSSSKIKAGAEQIK	transplant<br>blood donor NEG (BoDV-1)	3<br>2<br>(2)	endogenous Bornavirus like nucleoprotein 2	0.65	endogenous Bornavirus-like nucleoprotein 2 [Ictidomys tridecemlineatus]<br><br>endogenous Bornavirus-like nucleoprotein 1-like [Chrysochloris asiatica]	2E-09<br><br>3E-07

								endogenous Bornavirus-like nucleoprotein 1 [Mus caroli]<br><br>hypothetical protein B5V03_18725 [Bradyrhizobium betae]	0.092<br><br>0.51
N	41	161-175	QIKKRFKTMMAALNR	transplant	1	endogenous Bornavirus like nucleoprotein 2	0.28	endogenous Bornavirus-like nucleoprotein 2 [Ictidomys tridecemlineatus]	0.05
N	47-48	185-203	LQMENPHEAIDWINGQPWV	blood donor<br>neg ctrl<br>(BoDV-1)	1<br>1<br>(3)	endogenous Bornavirus like nucleoprotein 1	0.006	endogenous Bornavirus-like nucleoprotein 2 [Ictidomys tridecemlineatus]	2E-06
N	68-82	269-339	SAKLKEDHADLFPFLGAIRHPDAIKLAP<br>RSFPNLASAAFYWSKKENPTMAGYR<br>ASTIQPGASVKETQLARY	transplant<br>blood donor<br>blood donor NEG<br>(BoDV-1)	5<br>2<br>2<br>3	endogenous Bornavirus like nucleoprotein 1	2E-05	endogenous Bornavirus-like nucleoprotein 2 [Ictidomys tridecemlineatus]<br><br>endogenous Bornavirus-like nucleoprotein 1-like [Chrysochloris asiatica]<br><br>endogenous Bornavirus-like nucleoprotein 1 of several other species	5E-48<br><br>6E-21
X	1	1-15	MSSDLRLTLLELVRR	transplant	1	coiled-coil domain-containing protein 157	0.57	hypothetical protein C5Q97_08165 [Victivallales bacterium]	3.2
X	4	13-27	VRRLNGNATIESGRL	blood donor	1	NFIL3 like protein	3.2	AsmA-like C-terminal domain-containing protein	0.28

								[Deltaproteobacteria bacterium]	
X	5-7	17-35	NGNATIESGRLPGGRRRSPDTTT	transplant<br>blood donor<br>(BoDV-1)	1<br>1<br>2	gamma-taxilin	0.47	ABC transporter [Aeromicrobium sp.]	2.0
X	15-19	57-87	PTSRPAPEGPQEEPLHDLRPRPANRKGAAVE	transplant<br>blood donor<br>(BoDV-1)	4<br>2<br>(8)	germinal-center associated nuclear protein	1.1	hypothetical protein [Methylobacterium sp.]	0.99
P	1-5	1-31	MATRPSSLVDSLEDEEDPQTLRRERPGSPRP	transplant<br>blood donor<br>blood donor NEG<br>(BoDV-1)	3<br>1<br>1<br>(6)	PDCD7 protein<br><br>CDK5 and ABL1 enzyme substrate<br><br>CABLES	0.40<br><br>0.79<br><br>0.79	unnamed protein product [Mytilus coruscus]<br><br>hypothetical protein [Azospirillum ramasamyi]<br><br>Rab-GTPase-TBC domain [Phytophthora infestans]<br><br>neurotrophin receptor-interacting factor homolog [Chrysochloris asiatica]<br><br>tbc1 domain family member 23-like partial [Plasmopara halstedii]	0.067<br><br>0.19<br><br>0.26<br><br>0.72<br><br>0.72
P	9	33-47	KVPRNALTQPVDQLL	transplant<br>blood donor<br>blood donor NEG<br>(BoDV-1)	1<br>1<br>1<br>(4)	protein sprouty homolog 2	9.2	malto-oligosyltrehalose synthase [Proteobacteria bacterium]	6.3
P	11-13	41-63	QPVDQLLKDLRKNPSMISDPDQR	transplant<br>blood donor<br>(BoDV-1)	3<br>1<br>(6)	MLF1IP protein	0.91	methyltransferase [Pontibacter korlensis]	0.71

P	16-17	61-79	DQRTGREQLSNDELIKKLV	transplant<br>blood donor NEG<br>neg ctrl<br>(BoDV-1)	3<br>1<br>3<br>(4)	la-related protein 6	1.5	golgin subfamily B member 1 [Mugil cephalus]	0.78
P	19	73-87	ELIKKLVTELAENSM	transplant<br>blood donor<br>(BoDV-1)	1<br>1<br>(1)	zinc finger protein 469	6.5	sugar ABC transporter ATP-binding protein [Subtercola sp.]	6.3
P	23-24	89-107	EAEEVRGTLGDISARIEAG	transplant<br>(BoDV-1)	4<br>(2)	cytochrome P450 4X1	4.3	precorrin-4 C(11)-methyltransferase [Planctomycetota bacterium]<br><br>precorrin-4 C(11)-methyltransferase [Moorella sp.]	0.39<br><br>0.78
P	29-37	113-159	ALQVETIQTAQRCDHSDSIRILGENIKILDRSMKTMMETMKLMMEKV	transplant<br>blood donor<br>blood donor NEG<br>neg ctrl	5<br>1<br>2<br>1	alternative protein RBM28	2.7	hypothetical protein 3 [Bitis gabonica]<br><br>TPA: MMPL family transporter [Acholeplasmataceae bacterium]<br><br>TPA: GTPase HflX [candidate division Zixibacteria bacterium]<br><br>TPA: anion permease [Elusimicrobia bacterium]<br><br>MMPL family transporter [Acholeplasmataceae bacterium]<br><br>hypothetical protein [Cryomorpha ignava]	0.002<br><br>0.21<br><br>0.55<br><br>0.75<br><br>0.77<br><br>0.98

P	43	169-183	GTSAPMLPSHPAPPR	transplant (BoDV-1)	1 (3)	alternative protein KLC2	0.4	unnamed protein product [Taenia asiatica]<br><br>hypothetical protein F4802DRAFT_275747 [Xylaria palmicola]<br><br>Os09g0532600 [Oryza sativa Japonica Group]	0.56<br><br>0.79<br><br>0.8
P	48	189-203	QLPSAPTTDEWDIIP	blood donor (BoDV-1)	1 (4)	immunoglobulin heavy chain junction region	0.29	carboxypeptidase regulatory-like domain-containing protein [Deltaproteobacteria bacterium]	6.3

Peptides that were identified in a linear epitope mapping to be associated with false-reactive ELISA results were blasted against a human protein database and an organism-independent database (after exclusion of *Bornaviridiae*), respectively (https://blast.ncbi.nlm.nih.gov). All results with E values < 1 are given. For peptides with insignificant alignments (E value > 1), the lowest E value is shown.
